# Within-Host Evolution of *Burkholderia pseudomallei* in Four Cases of Acute Melioidosis

**DOI:** 10.1371/journal.ppat.1000725

**Published:** 2010-01-15

**Authors:** Erin P. Price, Heidie M. Hornstra, Direk Limmathurotsakul, Tamara L. Max, Derek S. Sarovich, Amy J. Vogler, Julia L. Dale, Jennifer L. Ginther, Benjamin Leadem, Rebecca E. Colman, Jeffrey T. Foster, Apichai Tuanyok, David M. Wagner, Sharon J. Peacock, Talima Pearson, Paul Keim

**Affiliations:** 1 Northern Arizona University, Center for Microbial Genetics and Genomics, Flagstaff, Arizona, United States of America; 2 Translational Genomics Research Institute, Phoenix, Arizona, United States of America; 3 Mahidol-Oxford Tropical Medicine Research Unit, Faculty of Tropical Medicine, Mahidol University, Bangkok, Thailand; 4 Department of Microbiology and Immunology, Faculty of Tropical Medicine, Mahidol University, Bangkok, Thailand; 5 Department of Medicine, University of Cambridge, Cambridge, United Kingdom; University of Toronto, Canada

## Abstract

Little is currently known about bacterial pathogen evolution and adaptation within the host during acute infection. Previous studies of *Burkholderia pseudomallei*, the etiologic agent of melioidosis, have shown that this opportunistic pathogen mutates rapidly both *in vitro* and *in vivo* at tandemly repeated loci, making this organism a relevant model for studying short-term evolution. In the current study, *B. pseudomallei* isolates cultured from multiple body sites from four Thai patients with disseminated melioidosis were subjected to fine-scale genotyping using multilocus variable-number tandem repeat analysis (MLVA). In order to understand and model the *in vivo* variable-number tandem repeat (VNTR) mutational process, we characterized the patterns and rates of mutations *in vitro* through parallel serial passage experiments of *B. pseudomallei*. Despite the short period of infection, substantial divergence from the putative founder genotype was observed in all four melioidosis cases. This study presents a paradigm for examining bacterial evolution over the short timescale of an acute infection. Further studies are required to determine whether the mutational process leads to phenotypic alterations that impact upon bacterial fitness *in vivo*. Our findings have important implications for future sampling strategies, since colonies in a single clinical sample may be genetically heterogeneous, and organisms in a culture taken late in the infective process may have undergone considerable genetic change compared with the founder inoculum.

## Author Summary

While both viral and bacterial pathogens have been shown to undergo genetic changes over the course of a chronic infection, this phenomenon has not been studied during an acute infection and as such is not well understood. Here, we examined within-host evolution of the pathogen *Burkholderia pseudomallei* during acute infection. *B. pseudomallei* causes the disease melioidosis, a significant cause of morbidity and mortality in many tropical regions of the world. We obtained multiple *B. pseudomallei* colonies from several tissue sites of four patients presenting with acute melioidosis in order to characterize how this bacterium evolves within the human host over a short period of time. By monitoring changes in rapidly evolving genetic regions, we found high levels of diversity of *B. pseudomallei* populations within a single patient, and even within a single body site. Comparison of these within-host mutation rates with *in vitro* mutation data enabled us to identify the most likely spatial migration of within-host populations and correlate these findings with clinical data to determine, in most cases, the origin of infection. Our study provides new insights into the evolution of bacterial pathogens during an acute infection, and lays the foundation for similar studies in other infectious agents.

## Introduction

*In vivo* studies of pathogen evolution have provided important insights into the dynamic adaptability of infectious agents during the course of an infection. To date, the overwhelming majority of within-host evolution work has focused upon monitoring pathogen adaptations concomitant with chronic infection establishment and persistence. In particular, *in vivo* population dynamics of human immunodeficiency-1 virus (HIV-1), the etiologic agent of acquired immunodeficiency syndrome (AIDS), have been intensely characterized (see [1]–[4] for salient examples). Due to their small genomes and high level of mutability, RNA viruses have provided an attractive avenue for investigating *in vivo* evolution of pathogen populations. Recently, *in vivo* studies of pathogen evolution have begun to shift towards bacterial infections. A hallmark study of *in vivo* bacterial evolution during chronic infection compared whole-genome sequences of two *Pseudomonas aeruginosa* strains derived from a single cystic fibrosis patient. Isolated 90 months apart, genetic changes conducive to niche adaptation and persistence within the complex lung environment were demonstrated [5]. However, despite these landmark studies, none have examined bacterial population diversity during an acute infection.

The Gram-negative bacterium *Burkholderia pseudomallei* is the cause of melioidosis, a potentially life threatening disease contracted through inhalation or direct inoculation of *B. pseudomallei* from contaminated soil or water [6]. Clinical manifestations and disease severity are highly variable. In its acute form, patients with melioidosis often present with bacteremia associated with bacterial dissemination, most often to the lung, liver and spleen [7],[8]. At over 7 Mbp and with two chromosomes, *B. pseudomallei* possesses one of the largest bacterial genomes characterized so far. Horizontal gene transfer, recombination and mutation all play a role in shaping its genome [9] and contribute to the impressive strain-to-strain variability observed in the pan-genome of *B. pseudomallei* [10],[11]. Within its genome are insertion sequence elements [10],[12], genomic island loci [11],[13],[14] and an unusually high number of variable-number tandem repeats (VNTRs) [10],[15]. Several studies have demonstrated that *B. pseudomallei* VNTRs can mutate over a short period of time. U'Ren and co-workers [15] conducted an *in vitro* parallel serial passage experiment (PSPE) of *B. pseudomallei* Bp9905-1902 and revealed large numbers of VNTR mutations upon short-term subculturing. Likewise, ten *in vivo B. pseudomallei* isolates collected over a two-week period from a single acute melioidosis patient were shown to harbor differences at VNTR loci [16]. Taken together, *B. pseudomallei* appears to possess the attributes required for rapid *in vivo* mutation and therefore micro-evolution over the period of an acute infection.

The aim of this study was to genetically characterize 182 primary agar plate colonies of *B. pseudomallei* isolated from multiple body sites from four Thai patients with acute melioidosis over a short period of time (two days to two weeks) using multilocus variable-number tandem repeat (VNTR) analysis (MLVA). The MLVA system targets 23 rapidly evolving repeat regions throughout the *B. pseudomallei* genome [15]. While not appropriate for examining more distant relationships due to its homoplastic nature, MLVA is appropriate for detecting genetic relationships amongst closely related isolates [16]. Our goals were two-fold; first, to assess the level of within-host genetic variation at different body sites and to examine whether genotyping could identify the founder genotypes and suggest the primary site of clinical infection and routes of dissemination based upon the MLVA patterns, and second, to model *in vivo* mutations using observed *in vitro* VNTR mutation rates and to make inferences about the spatial distribution of *B. pseudomallei* in acute melioidosis infections.

## Results

### Genotyping of within-patient isolates

We applied pulsed-field gel electrophoresis (PFGE) to all 182 isolates obtained from the four patients to determine clonality of infection. PFGE has previously been used to rule out re-infection from a different *B. pseudomallei* strain or simultaneous infection with multiple strains [17],[18], and is commonly used to differentiate closely related bacterial strains within outbreaks [19]. In the current study, PFGE demonstrated that *B. pseudomallei* genotypes were monomorphic within each patient (results not shown). While this method did not provide high resolution within an infection, the clonality of pulsotypes suggested that all isolates within each patient arose from a single cell or population of clonal *B. pseudomallei*. MLST of two primary colonies from each patient supported the homogeneity of within-patient genotypes (STs 670, 208, 177 and 671 for patients 19, 23, 44 and 45, respectively). STs 670 and 671 were novel whereas STs 177 and 208 have been identified previously in Thai melioidosis patients.

**Figure 1:**
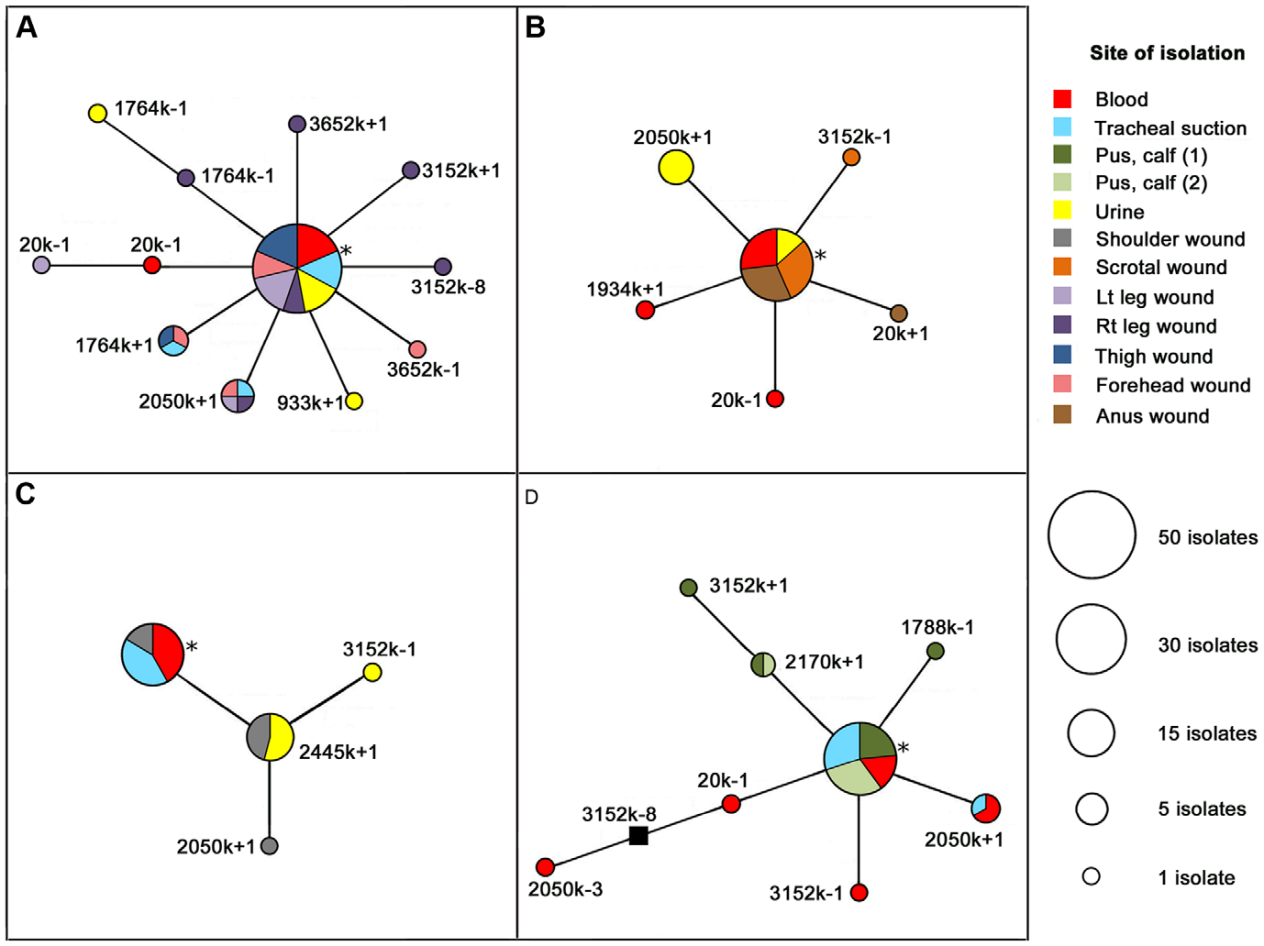
Maximum parsimony phylogenies of *Burkholderia pseudomallei* isolates derived from four Thai acute melioidosis patients. Phylogenies for patients 19, 23, 44 and 45 are shown in Panels A-D, respectively. Multiple tissue sites from each patient were cultured (e.g. blood, urine, tracheal, wound and pus samples). Where possible, ten colonies from each tissue site were retrieved; isolates were genotyped using a 23-locus multilocus variable-number tandem repeat analysis. In each patient, the numerically dominant genotype was assumed to be the original infecting strain. Specific mutations are displayed alongside each branch (e.g. 3652k-1 refers to a one-repeat deletion at locus 3652k). A single, theoretical, intermediate genotype not observed in our isolate set (Panel D, 20k+1 and 3152k-8) is represented by a solid black square. Asterisks indicate probable founder genotypes. See Figures S1 and S2 for alternative P19 and P45 phylogenies and Table S2 for *in vivo* mutation rate calculations.

Unlike PFGE and MLST, the higher-resolution MLVA technique discriminated among within-patient isolates. In addition to confirming clonality of the four acute infections, MLVA provided discrimination amongst the otherwise indistinguishable isolates from within each patient, with VNTR mutants being detected at multiple tissue sites. Due to the presence of multiple disseminated small abscesses, P19 had the greatest number of sampled tissue sites (*n* = 7), primary colonies (*n* = 65) and MLVA genotypes (*n* = 12). Between 37 and 40 primary colonies were obtained from four tissue sites in the other three patients and a total of eight, six and four MLVA genotypes were identified in P45, P23 and P44, respectively (Figure 1).

### *In vitro* mutation rates at VNTR loci

An *in vitro* parallel serial passage experiment (PSPE) for the genome-sequenced Bp305 strain was undertaken in order to quantify *in vivo* VNTR mutation patterns, and to add to our knowledge of *in vitro* VNTR mutation rates in *B. pseudomallei*. A summary of the *in vitro* 23-MLVA mutation rates for the Bp305 PSPE alone and in combination with a previous PSPE for Bp9905-1902 is shown in Table 1. Thirty-one mutations were observed in Bp305 at T_10_, compared with 12 mutations in Bp9905-1902 at T_10_. In Bp9905-1902, an overwhelming number (95%) of these mutations consisted of single-repeat changes with only a single T_10_ mutant characterized by a multi-repeat change [15]. Bp305, on the other hand, demonstrated multi-repeat changes in approximately 40% of mutations, most of which occurred at locus 2170k (Table 1). Bp9905-1902 mutated at eight of the 23 VNTR loci whereas Bp305 only mutated at five, despite the inherent larger number of generations in the Bp305 PSPE due to its longer subculturing time (24 and 48h, respectively). This difference could be attributable to the larger repeat copy numbers of the additional mutating loci in Bp9905-1902 compared with Bp305, as has been previously observed amongst *Escherichia coli* strains [20]. A high number of PCR failures were evident in the T_10_ Bp305 clones for the 20k (48%) and 2356k (33%) loci, as previously observed in certain VNTR loci of Bp9905-1902 [15]; these failures are probably a consequence of mutation at the primer binding sites or recombination, resulting in an insertion or deletion that would affect PCR efficiency. Given these high failure rates there is reduced potential for discovering mutations at these loci.

**Table 1:** *In vitro* mutation rates for 23 *Burkholderia pseudomallei* multilocus variable-number tandem repeat analysis loci.

Locus	Repeat motif (bp)	Mutation rate (Bp9905-1902 and Bp305 combined*)	Repeat copy no. (Bp305)	Mutation rate (Bp305)	Repeat copy no. (Bp9905-1902)**	Mutation rate (Bp9905-1902)**	Bp305 total no. mutations	Bp305 no. lineages	Bp305 no. insertions	Bp305 no. deletions	Bp305 no. single repeat changes	Bp305 no. multi-repeat changes
2170k	9	4.71×10^−4^	41	6.28×10^−4^	13	2.28×10^−4^	17	99	3	14	9	8
2050k	9	1.97×10^−4^	18	2.93×10^−4^	17	5.45×10^−5^	8	100	3	5	6	2
3152k	6	1.31×10^−4^	17	1.48×10^−4^	24	1.06×10^−4^	4	99	3	1	3	1
3145k	9	2.28×10^−5^	3	---	14	6.05×10^−5^	0	100	---	---	---	---
933k	12	3.66×10^−5^	8	---	14	5.51×10^−5^	0	100	---	---	---	---
1788k	16	2.20×10^−5^	7	---	9	5.45×10^−5^	0	98	---	---	---	---
2815k	9	2.17×10^−5^	6	---	30	5.28×10^−5^	0	99	---	---	---	---
20k	7	3.02×10^−5^	12	---	17	5.28×10^−5^	0	52	---	---	---	---
2065k	8	2.25×10^−5^	11	3.93×10^−5^	17	---	1	93	1	0	1	0
1764k	12	2.20×10^−5^	19	3.77×10^−5^	10	---	1	97	0	1	0	1
Average		9.77±1.4×10^−5^		1.92±0.24×10^−4^		1.59±2.4×10^−4^						
Total	---	∑ = 9.77×10^−4^	---	∑ = 1.15×10^−3^	---	∑ = 7.70×10^−4^	31	---	10	21	19	12
Percent	---	---	---	---	---	---	---	---	32.3	67.7	61.3	38.7

PSPE, parallel serial passage experiment.

*includes instances where one strain did not have any observed mutations for a given locus.

**data from [15] were rescored in the current study and edited accordingly.

Thirteen of the 23 MLVA loci did not mutate in either the Bp305 or Bp9905-1902 PSPEs, three of which (3652k, 2445k and 1934k) had novel mutations in our *in vivo* patient isolates. For these non-mutated loci, the limit of detection for the combined PSPE data (2.4×10^−6^ mutations/generation) was used to calculate the probabilities of phylogenies for P19 and P45 (Figures S1 and S2), since the likely mutation rate is near to or less than this rate. The single-locus mutation rates in the combined Bp305 and Bp9905-1902 *in vitro* dataset ranged from 2.17×10^−5^ to 4.71×10^−4^ mutations/generation, with an average mutation rate of 9.8±1.4×10^−5^ mutations/generation (Table 1). This rate is greater than the average VNTR mutation rate at 43 loci for *Y. pestis* (6.3±0.09×10^−5^ mutations/generation) [21],[22] and the 28-locus rate for *E. coli* O157:H7 (3.8±0.10×10^−5^ mutations/generation) [20]. This rate confirms previous reports demonstrating very high VNTR mutation rates in *B. pseudomallei* [15],[16],[23].

### VNTR mutation modeling in *B. pseudomallei*

**Figure 2:**
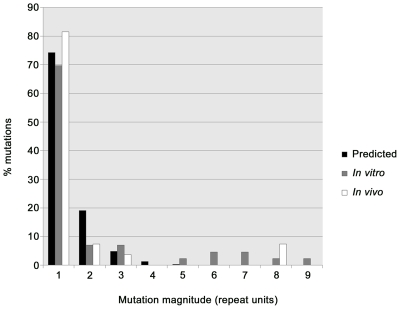
Frequency distribution of *Burkholderia pseudomallei* multilocus variable-number tandem repeat (VNTR) analysis mutations. The predicted geometric distribution model (black) was compared with *in vitro* (gray) and *in vivo* (white) VNTR mutation data. The geometric distribution model fits well with observed mutations of between one and four repeats; however, this model is not appropriate for predicting 5+ repeat copy number mutations, as previously observed in *Escherichia coli* O157:H7 [20].

We compared the patterns of *in vitro* and *in vivo* VNTR mutations observed in the combined PSPEs and the four acute melioidosis patients, respectively, with the patterns expected based upon a theoretical model. Vogler and co-workers have proposed a general VNTR mutation model describing the distribution of VNTR mutations involving different numbers of repeats, and have demonstrated its applicability for modeling VNTR mutation patterns in *E. coli* and *Y. pestis* [20],[22]. We used this model and an observed frequency of 74.3% single-repeat mutations (comprising both insertions and deletions) in our combined PSPEs to construct a theoretical distribution of *B. pseudomallei* VNTR mutations (Table 1; Figure 2). While the distribution predicted from this model fitted well with the observed number of mutations comprising one-, two-, three- and four-repeats in the combined PSPEs, there were more observed mutations involving greater than four repeats than expected based upon the theoretical model (Figure 2); this circumstance has also been observed in a series of *E. coli* O157:H7 PSPEs [20]. Several loci (2050k, 3152k, 2170k and 1764k) had multi-repeat deletions in the Bp305 PSPE, with 2170k exhibiting an elevated number of multi-repeat deletions compared with other loci, an expected finding given the large number of repeats in Bp305 at this locus compared with Bp9905-1902 (Table 1). A similar large VNTR locus (O157-10) in *E. coli* O157:H7 PSPEs, which ranged from nine to 66 repeats, contributed to elevated mutation rates in this species [20]. There was a predominance of multi-repeat deletions over insertions in our dataset, particularly at 2170k, although both are theoretically equally likely; this trend was also observed in *E. coli* and has been hypothesized to be a result of positive selection favoring deletions in large arrays [20].

We characterized earlier time points for all Bp305 lineages containing multi-repeat VNTR mutations using MLVA to identify whether these mutations occurred as single or multiple mutation events (Table S1). The mechanism behind multi-repeat VNTR mutation events is important in determining the probability of the mutation, as both slipped-strand mispairing and recombination have been suggested as mechanisms of multi-repeat VNTR mutations and the expected distribution of mutation types for these two mechanisms is considerably different [20],[24]. Importantly, recombination events leading to multi-repeat mutations cannot be modeled accurately using the geometric distribution VNTR model, as this model assumes that only slipped-strand mispairing causes VNTR mutations. By analyzing intermediate generations (backtracking) using glycerol stocks from our Bp305 PSPEs, we were able to ascertain that all of the *in vitro* multi-repeat deletions likely occurred in a single evolutionary step (Table S1) and, as such, were most likely due to single recombination events rather than large slipped-strand mispairing events. Removal of 2170k, the locus with the greatest number of multi-repeat mutations, from the PSPE data yielded a mutation type distribution that more closely mimicked the expected slipped-strand distribution (results not shown). Due to the likely involvement of recombination in generating multi-repeat mutations greater than four repeats *in vivo* (i.e. the 3152k eight-repeat mutation occurrences in P19 and P45) the probability of these events are grossly underestimated by the geometric distribution model (Figure 2). Nevertheless, we consider the geometric distribution to be suitable for modeling *in vivo* mutations that involve one, two, three or four repeat mutations in *B. pseudomallei*, consistent with the *E. coli* O157:H7 PSPEs [20].

### *In vivo* phylogenetic modeling

Maximum parsimony phylogenies for the within-host isolates from each patient (Figure 1) were evaluated using a combination of *in vitro* mutation rates (Table S2) and the geometric distribution VNTR model. For P23 and P44, a single possible phylogeny was constructed (Figure 1B and C), whereas multiple parsimonious phylogenies were constructed for P19 and P45. Comparison of alternative P45 phylogenies using the odds ratio approach suggested that the phylogeny in Figure 1D was much more likely than other phylogenies (ORs>150; Figure S2). In contrast, the P19 phylogeny shown in Figure 1A was only slightly more likely than alternative phylogenies (ORs<20), which were therefore considered equally parsimonious (Figure S1).

### Founder genotypes and subgroup founders

Identifying the founder genotype enables a better understanding of *in vivo* spatial distribution of genotypes and potential routes of infection. The founders were initially defined using eBURST, a widely implemented algorithm that assesses divergence from a founder based upon parsimony principles [25]. eBURST calculates the founder as the genotype with the greatest number of single-locus, and when identical, double-locus variants. In recently diverged populations such as clonally derived *in vivo* populations, the founder would also be expected to be numerically dominant.

We observed that the eBURST founders were both numerically dominant and were disseminated to the greatest number of tissue sites in P19, P23 and P45, with eBURST bootstrap values of 100, 97 and 94%, respectively (Figure 1A, 1B and 1D). In contrast, eBURST analysis of P44 isolates did not identify the numerically dominant and most spatially distributed genotype as the founder genotype. Instead, eBURST assigned the 2445k+1 genotype (urine and shoulder wound samples) as the founder due to this genotype exhibiting the greatest number of single-locus variants (Figure 1C). Unlike in the other patients, this P44 founder assignment was not strongly supported by eBURST, with only 72% founder bootstrap support. As the eBURST algorithm does not take into account spatial distribution of genotypes, we assigned the genotype with the greatest frequency and degree of spatial distribution as the P44 founder (Figure 1C). We assumed that the P44 2445k+1 genotype was instead a subgroup founder (a clone that has diversified and spawned its own clones) of the larger ‘founder’ population.

### Comparison of clinical features and disease progression with MLVA data

It was possible to pinpoint the probable site of the initial infection in some of the patients based upon the tissue site exhibiting the greatest degree of genetic diversification from the founder genotype. For instance, the MLVA data indicate that the probable primary site of initial infection in P44 and P45 are the shoulder and calf wounds, respectively (Figure 1C and 1D), consistent with clinical observations (Table 2). The primary site of infection of P23 is more difficult to define as, according to the MLVA data, both the anal and scrotal abscesses are likely, and possibly simultaneous, candidates (Figure 1B). This observation is in agreement with the clinical data, which indicated that this patient had two weeks of progressive anal and scrotal abscesses (Table 2).

**Table 2:** Within-patient Thai *Burkholderia pseudomallei* isolates used in this study.

Patient ID	Specimen location	Collection date	No. colonies	Proportion of founder genotypes	Proposed site of primary infection*	Clinical features and outcome
P19	BloodTracheal suctionUrinePustule, right legPustule, left legPustule, foreheadWound swab, thigh	17-Jul-0617-Jul-0617-Jul-0617-Jul-0617-Jul-0617-Jul-0618-Jul-06	1099	9/107/97/94/98/105/89/10	x	22 y.o. male, admitted after a motorcycle accident. Day 4 developed necrotizing fasciitis with abscess formation in a left thigh wound from which *Streptococcus pyogenes* was isolated. Blood culture on day 6 was positive for *B. pseudomallei* (not available for this study). Samples shown here were taken on day 11 & 12 when the patient had disseminated melioidosis with bacteremia, multiple cutaneous abscesses and pneumonia. The left thigh wound was positive for *B. pseudomallei* at this stage. Patient died from fulminant sepsis.
P23	UrinePus, right scrotal abscessPus, anal abscessBlood	20-Jul-0620-Jul-0620-Jul-0620-Jul-06	10101010	4/109/109/108/10	xx	60 y.o. diabetic male rice farmer presented with 14 day history of progressive perianal and scrotal abscesses. Blood and abscess material obtained on day 1 was positive for *B. pseudomallei* (not available for this study). Samples shown here were taken on day 4 when the patient had disseminated melioidosis. Patient died from fulminant sepsis.
P44	Tracheal suctionBloodUrineAbscess, left shoulder	2-Aug-062-Aug-062-Aug-062-Aug-06	1010710	10/1010/100/74/10	x	34 y.o. female rice farmer presented with a five-day history of left shoulder abscess. Abscess pus taken on admission was positive for *B. pseudomallei*. Samples shown here were taken on day 3 when the patient had disseminated melioidosis. Patient died from fulminant sepsis.
P45	Pus, calf (1) aIsolator (blood)Pus, calf (2)Tracheal suction	1-Aug-062-Aug-062-Aug-0620-Aug-06	10101010	7/105/109/109/10	x	51 y.o. male rice farmer presented with 7 day history of right calf abscess; two pus samples and blood culture were positive for *B. pseudomallei* (samples shown). Pneumonia developed on day 19 despite appropriate antimicrobial therapy when a tracheal suction sample was positive for *B. pseudomallei* (samples shown). Patient taken home by family; outcome unknown.

*According to clinical data.

a Isolates collected from the same tissue site but at different times.

Patient 19 developed disseminated melioidosis following a motorcycle accident that resulted in a head injury, left thigh wound and multiple abrasions. Clinically, the most likely site of primary infection was the left thigh, which progressed to necrotizing fasciitis (and was later positive for *B. pseudomallei*), although entry of *B. pseudomallei* via the multiple abrasions and inhalation cannot be excluded. Subsequent bacterial dissemination after a period of around one week from the initial thigh wound infection or an alternative, clinically covert focus was associated with positive blood cultures, pneumonia and multiple subcutaneous abscesses. The MLVA profile for 65 colonies from seven sampling sites demonstrated approximately equal levels of genetic divergence from the founder genotype at multiple sites (Figure 1A and Table 2).

Patients 23, 44 and 45 had similar clinical presentations, with a history of an abscess as the primary site of infection followed by bacterial dissemination to other sites in association with clinical deterioration. Patient 23 presented with a two-week history of perianal and scrotal abscesses, and had disseminated melioidosis on admission with cultures positive from blood and urine in addition to positive abscess cultures (Table 2). The MLVA data based on a total of 40 colonies from four samples (Figure 1B) indicated that the founder genotype was present and predominated in the two abscesses as well as in urine and blood, with genetic diversification in all samples. Patient 44 presented with a five-day history of an abscess at the left shoulder, followed by subsequent bacterial dissemination. MLVA data based on a total of 37 colonies from four samples (Figure 1C) indicated that the putative subgroup founder (2445k+1) was present in the shoulder abscess and urine, whereas the putative founder was identified in the shoulder, blood and lungs. Patient 45 initially presented with acute symptoms (fever and calf abscess) with defervescence five days post-ceftazidime treatment. However, this patient experienced relapse of fever accompanied by respiratory distress approximately two weeks after the *B. pseudomallei-*positive pus and blood specimens were collected, at which time the tracheal suction isolates from P45 were obtained. These temporally distinct tracheal suction isolates demonstrated the persistence of the founder genotype over this two-week period (Figure 1D). In addition to the founder genotype, a single 2050k+1 mutant was identified in both the tracheal suction isolates and in the blood, but not in the pus samples. These P45 results indicate the persistence of the founder genotype andthe 2050k+1 genotype in the lungs. However, greater numbers of isolates from several tissue sites, including later samples from blood and calf abscess, would be required to confirm this hypothesis.

Interestingly, the P44 founder genotype was not observed among the urine-derived isolates, suggesting potential independent spread of the subgroup founder from its point of origin (i.e. the shoulder wound) to the renal system. A similar subgroup founder in P45 (2170k+1) showed site-specific diversification of calf-derived pus samples. Other potential subgroup founders were identified in P19 (Figure 1A, 20k-1 and 1764k-1) and P45 (Figure 1D, 20k+1), although only a single representative was observed. While no subgroup founders were found in P23, the urine isolates were mostly of a novel genotype, represented by a single repeat insertion at 2050k, with only four of the ten urine isolates sharing the founder genotype. Unlike the P44 urine-derived isolates, no other tissue sites shared the dominant P23 urine genotype (Figure 1B, 2050k+1).

## Discussion

Previous studies defining the proportion of patients with melioidosis that are infected with more than one strain of *B. pseudomallei* during a single infective episode have utilized PFGE or ribotyping to characterize multiple *B. pseudomallei* colonies from the same patient [17],[26]. While these methods are useful for identifying polyclonal infections, they cannot discriminate among multiple colonies from patients infected with a single strain, as they lack sufficient resolution. These methods are also not definitive indicators of monoclonal infection, particularly if environmental diversity is limited. In a recent study, MLVA was used to characterize ten *B. pseudomallei* colonies from a single patient with acute melioidosis [16]. The current study sought to expand on this previous work by characterizing *in vivo* changes in VNTR loci using 182 *B. pseudomallei* obtained from several body sites from four patients with severe acute melioidosis. MLVA provided important insights into the genetic heterogeneity of individual strains of *B. pseudomallei* by offering fine-scale resolution unattainable using MLST and PFGE, which, similarly to previous studies, were unable to differentiate between within-patient colonies. Indeed, even current next-generation whole genome sequencing (WGS) technologies may prove unfruitful in achieving the resolution of the 23-MLVA approach, due to inherently poor characterization of repeat regions [27], in which much of the genetic variation within this bacterial species is likely to be missed. Additionally, it is currently impractical to perform WGS on such a large number of isolates. As WGS becomes cheaper and more mature, it will be capable of capturing both fine and large scale evolution and will supersede our MLVA approach. In the interim, we have demonstrated that MLVA is a powerful method for defining fine-scale epidemiological patterns over short-term *in vivo* passage.

The pathophysiology of melioidosis is poorly understood due to the frequent lack of obvious inoculation site or recent trauma, the broad and inconsistent nature of symptoms between individuals, and the fact that almost any body site can become involved during an infection [28],[29]. Compounding this difficulty, differences in disease characteristics are likely conferred by both host and pathogen factors. As genetic plasticity is a hallmark of the *B. pseudomallei* genome, one of our goals was to better understand the extent to which the infecting isolate underwent genetic change *in vivo*. In addition, we wanted to compare the genetic changes identified by MLVA against detailed clinical features to determine the ability for MLVA to predict the primary site of inoculation. There was general concordance between clinical and genetic approaches for three of the four melioidosis patients (P23, P44 and P45), all of whom developed one or more clinically defined and circumscribed abscesses prior to bacterial dissemination and clinical deterioration. Dissemination of the founder genotype was observed in these three patients, with genetic variants identified both in the primary infection focus and in putatively seeded sites. On the other hand, P19 had a more complex clinical history that was suggestive of infection of a thigh wound inflicted during a motorbike accident. While the thigh wound was the most plausible primary focus, the clinical data were unable to exclude simultaneous *B. pseudomallei* inoculation of numerous superficial epidermal abrasions. By applying PFGE and MLST to the P19 isolates, it was demonstrated that multiple inoculation events were unlikely given the monoclonal nature of the infection. Using MLVA, we obtained approximately equal levels of divergence from the founder genotype, and it was not possible to identify a tissue site consistent with the primary site of infection. The MLVA data for P19 suggested two hypotheses; firstly, that the founder genotype present in the primary inoculation site (e.g. the thigh wound) may have disseminated to numerous secondary sites prior to subsequent genetic diversification. Alternatively, an inhalation event may explain founder genotype dissemination to multiple tissues. However, an inhalational route of exposure is inconsistent with clinical progression in P19, as inhalational melioidosis in Thai patients is commonly associated with fulminant pneumonia within 24 hours of exposure [30]. Therefore, the MLVA pattern and clinical data for P19 are suggestive of dissemination of the founder genotype following primary inoculation of a single focus (i.e. thigh wound) of infection. The complexity of the MLVA pattern in this patient may also be a function of the greater number of tissue sites, and hence colonies, obtained from this patient. To resolve the precise route of infection with MLVA, earlier isolates from multiple tissue sites, especially from the necrotizing thigh wound, would have been necessary. Taken together, these data demonstrate that both clinical and genotype data can be used to determine the site of primary infection, but also highlight the need for even more intense sampling of tissue sites over multiple time periods in complex clinical cases.

We observed an interesting pattern in the urine MLVA genotypes derived from P23 and P44, which were predominantly genetically distinct from their putative founder populations. Six out of ten urine isolates from P23 had a genotype that was not observed in other tissues. Similarly, the seven urine isolates from P44 comprised two genotypes, one of which was not found in other tissues. It is unclear why the urine isolates were distinct from the founder genotype in these patients. These urine-derived genotypes may have migrated from unsampled organs and tissue sites, or may indicate tissue compartmentalization (segregation) of *B. pseudomallei* in the renal system. For example, the 2445k+1 genotype (urine and shoulder isolates) in P45 may have arisen following transit of a shoulder wound isolate to the renal system via the bloodstream. The possibility of independent growth in the urinary tract system after segregation is supported by the lack of correlation between counts of the organism in blood and urine [31]. An alternative hypothesis for these segregated MLVA profiles is that the infecting inoculum contained multiple genotypes, and a secondary genotype was more fit for survival in the kidneys; however, the identical PFGE patterns observed for all isolates from P23 and P44 does not support this alternate hypothesis. Therefore, the renal system may play a more significant role in melioidosis pathogenesis than presently appreciated. Unfortunately, due to the poor outcomes of the acute melioidosis patients from this study, it was not possible to collect temporal isolates from these three patients. Further studies using greater numbers of temporal isolates from multiple tissue sites are needed to test the compartmentalization hypothesis of urine samples.

Patient 45 was the only patient in our study that afforded temporal comparison of genotypes *in vivo.* Isolates from a tracheal suction were collected two weeks after the initial isolates were obtained, following the development of pneumonia despite antimicrobial therapy. The MLVA results indicated that the tracheal isolates mostly harbored the founder genotype, but also shared a second genotype with blood isolates collected two weeks earlier, suggesting not only persistence of the founder genotype throughout the course of the infection but the possibility of seeding of *B. pseudomallei* into the lungs (via the bloodstream) from an unsampled tissue site. Potential tissue compartmentalization was also observed in P45, where calf abscess samples appeared to segregate from blood and tracheal samples (Figure 1D). As in P23, we did not find evidence for multiple genotypes in the infecting inoculum, which is based upon our observation of identical PFGE profiles from all P45 isolates. This segregation of MLVA genotypes was an unexpected finding that suggests potential independent evolution of the calf abscess isolates and the contribution of an auxiliary, unsampled tissue site (e.g. the spleen) to bloodborne, and ultimately lung, diversity.

In order to understand *in vivo* mutation rates and patterns, we undertook an *in vitro* mutation experiment of Bp305 and combined these data with a previous PSPE on Bp9905-1902 [15]. While one-, two- three- and four-repeat mutations appeared to fit well with a general VNTR mutation model [20], the likely contribution of recombination in generating *B. pseudomallei* VNTR mutations comprising five or greater repeats rendered it difficult to accurately model multi-repeat mutations *in vitro* and *in vivo*. This difficulty is exemplified by estimates of the number of generations required to attain the eight-repeat deletion at locus 3152k, a mutation observed *in vivo* in both P19 and P45. According to the general VNTR mutation model and our *in vitro B. pseudomallei* VNTR mutation rates, the 3152k-8 event would be expected to occur every 1.4×10^8^ generations (95% confidence interval: 3.5×10^6^ to 7.4×10^8^ generations; Table S2); however, given the short time scale of infection, this number of generations is not biologically possible. As such, it is tempting to speculate that the *in vivo* mutation rate is potentially much greater than witnessed *in vitro*; however, the involvement of recombination events occurring at unknown rates, as well as three subculturing events prior to *in vivo* isolate characterization, complicates any such conclusions. While we were unable to directly compare the relative rates of *in vitro* and *in vivo* mutation due to the use of different strains from the patients and in the PSPEs, future PSPEs on the *in vivo* isolates could shed light on potential locus-based differences in these strains and may more accurately model the *in vivo* mutation events described in this study. The combined PSPE data will prove useful in future *in vivo* evolution studies of *B. pseudomallei*, such as for determining *in vivo* generation times in temporally-related isolates.

The 23-MLVA markers, which are predominantly intergenic and thus presumably selectively neutral, represent only a fraction of the loci that contribute to *B. pseudomallei* diversity. VNTRs not used in our 23-MLVA scheme, including those in coding regions or VNTRs that affect transcription, are important for understanding bacterial fitness in different niches within the human host. Analysis of other genetic markers encoding virulence factors, antimicrobial resistance, or genes involved in enhanced invasion, evasion and mutability would shed insights into pathogen adaptability under immune and drug pressures and could be observed using WGS. For instance, horizontal gene transfer within genomic islands that encode proteins involved in virulence, tissue tropism, and persistence probably influence ecological establishment and clinical outcome [11],[13],[14]. Sam *et al*. [32] recently demonstrated heterogeneity in antibiotic susceptibilities of a small number of isolates from a single patient. Our large collection of *in vivo* isolates provides an opportunity to expand on the Sam *et al.* study and build upon our current limited knowledge in this field of melioidosis research. Other avenues of investigation using an *in vivo* evolution approach include examining the bacterial factors behind chronic persistence of melioidosis, or the involvement of co-infections, such as the group A *Streptococcus* co-infection observed in P19, in generating genetic diversity in *B. pseudomallei* during acute and chronic melioidosis.

It is recognized that there are potential criticisms of our approach. Firstly, only a maximum of ten colonies per tissue site were obtained, resulting in sampling bias towards more dominant genotypes and potentially overlooking minor genotypes within samples. Secondly, there are known differences in *B. pseudomallei* growth rates and population size between different anatomical sites. We sampled essentially identical numbers of colonies from each anatomical site and did not take into account the inherently higher genetic variation at a high bacterial load (e.g. respiratory secretion and pus) versus a low bacterial load site (e.g. blood) [33]. Such differences in replication rates may have an impact on the prediction of the founder genotype. While we used eBURST principles to predict the founder (i.e. the most numerically dominant genotype with the greatest number of single-locus variants), more detailed analyses are required to verify these founder assignments, such as animal model studies or phylogenetic outgroup rooting with a closely related environmental isolate. Thirdly, the identification of anatomical sites with low prevalence of the founder genotype, such as observed in the P23 and P44 urine sites, could feasibly arise by chance. Unfortunately, we were unable to apply meaningful confidence intervals to our small dataset to determine whether the low prevalence of founder genotypes in the urine-derived isolates from P23 and P44 were significant, and therefore we cannot rule out stochastic factors contributing to these observations. Lastly, many samples were taken several days after onset of symptoms and sampling time points were not consistent across the patient group. Despite these shortcomings, this study is the first of its kind to explore acute infection in such depth and many of these issues were not known at the commencement of the study. Based upon our observations, we recommend that future work in this field should attempt to reduce the effects of genotype bias by using greater numbers of isolates from a single tissue site, including larger numbers of study patients, and aiming for the collection of temporal isolates from the same tissue site/s, in order to more rigorously test *in vivo* pathogen evolution hypotheses.

In conclusion, the current study is the first to employ fine-scale genotyping on a comprehensive *in vivo* isolate collection derived from acute infections. Given the remarkable genetic diversity of *B. pseudomallei* within a single tissue site at a single time point, this investigation highlights the rapid genetic modification and potential for adaptability of *B. pseudomallei* to niches within the human host. The ability for *B. pseudomallei* to diversify over a short time frame has substantial implications for our understanding of treatment, eradication and prevention of melioidosis. Our findings are not only of interest to melioidosis researchers, but will likely have major impact in the food safety, clinical and microbial forensic communities. For example, the use of limited numbers of reference strains in an epidemiological context should be questioned in light of our study. We recommend that sampling and genetic characterization of several isolates from both the source and the host, or carrier, becomes the standard for such investigations in order to account for inevitable evolutionary processes.

## Methods

### Ethics statement

Informed written consent has previously been obtained from each subject enrolled into the study [17].

### Study subjects and specimen processing

The four study subjects with acute melioidosis described herein (patients 19, 23, 44 and 45) represent a subset of patients recruited into a prospective study that defined rates of polyclonal *B. pseudomallei* infection. Patients were admitted between June and August 2006 to a hospital in Ubon Ratchathani, northeast Thailand [17]. In brief, patients with suspected melioidosis were sought during twice-daily ward rounds of the medical and intensive care wards and multiple samples were taken from suspected cases. A 15mL blood sample was taken and divided between a BacT/ALERT® FA bottle (BioMérieux, Durham, NC) for standard culture (5mL), and an Isolator 10 lysis centrifugation tube (Oxoid, Basingstoke, Hampshire, UK) (10mL). Samples were taken for culture of sputum/tracheal aspirate, throat swab, urine, pus or surface swab from wounds and skin lesions as appropriate.

The objective of our culture techniques was to achieve single *B. pseudomallei* colonies on primary agar plates that had been directly inoculated with the clinical sample [17]. Briefly, clinical specimens were spread-plated onto Ashdown agar [34]. Colonies suspected to be *B. pseudomallei* were tested using an oxidase test and subsequently confirmed with a *B. pseudomallei*-specific latex agglutination test [17]. Ten primary plate colonies were picked from each sample positive for *B. pseudomallei* and saved to independent freezer vials; all colonies were picked if fewer than ten were present. Each colony was stored at −80°C and sub-cultured a total of three times prior to DNA extraction for MLVA.

The four patients were selected from patients with four or more samples positive for *B. pseudomallei*. The number of primary colonies per patient ranged from 37 to 65, and the total number tested was 182. The clinical history of the four cases is summarized in Table 2. All four patients had disseminated melioidosis. Three patients died in hospital, and the fourth was seriously ill when taken home by relatives and his outcome is unknown.

### DNA isolation

Genomic DNA (gDNA) was extracted from clinical *B. pseudomallei* isolates propagated on Luria-Bertani agar (Becton Dickinson, Franklin Lakes, NJ) for 48 h at 37°C using the DNeasy™ 96 Tissue Kit (Qiagen Inc., Valencia, CA) according to the manufacturer's protocol. A 5% chelex-100 (BioRad, Hercules, CA) extraction protocol [35] was used to isolate gDNA from *B. pseudomallei* 305 (Bp305) PSPE isolates (see ‘*In vitro* mutation rates of VNTRs in *B. pseudomallei*’ below for isolate descriptions). Qiagen-extracted gDNA was quantified fluorimetrically (SpectraMax Gemini; Molecular Devices, Sunnyvale, CA) and diluted to 500 pg/µL in molecular grade H_2_O (Invitrogen, Carlsbad, CA); chelex-extracted gDNA was diluted 1∶9 in molecular grade H_2_O.

### Genotyping

Multilocus sequence typing (MLST) was undertaken on two primary colonies from each of the four patients. MLST was performed according to Godoy *et al*. [36]. To improve amplification robustness for the *gmhD* locus, we used modified amplification primers [18]. New sequence types (STs) were submitted to the *B. pseudomallei* MLST database (http://bpseudomallei.mlst.net/). Pulsed-field gel electrophoresis was performed on all 182 primary plate colonies, as previously described [18].

MLVA based on 23 predominantly intergenic, and hence presumed to be selectively neutral, VNTR loci was performed on all colonies as previously detailed [15],[23], with the exception of an increase in betaine (Sigma-Aldrich, St Louis, MO) concentration in the multiplex PCRs from 1.2 to 2.0M. Briefly, MLVA involved multiplexed PCR amplification of VNTR regions using differentially labeled fluorescent primers. Following amplification, the PCR products were diluted 1∶25 in molecular grade H_2_O, denatured in HiDi™ formamide (Applied Biosystems, Foster City, CA) and subjected to capillary electrophoresis using a 3730*xl* DNA Analyzer (Applied Biosystems). Size determination was carried out relative to an internal LIZ1200® ladder (Applied Biosystems) and GeneMapper version 4 software (Applied Biosystems) was used for peak analysis [15],[16]. All data were independently double-scored by two researchers.

### *In vitro* mutation rates of VNTRs in *B. pseudomallei*

A ∼27,000 generation parallel serial passage experiment (PSPE) was carried out to determine *in vitro* mutation rates for the 23 VNTR loci used in the MLVA. Such estimates can be used to facilitate phylogenetic analyses of isolates cycling *in vivo* [22]. While a PSPE has been carried out on *B. pseudomallei* Bp9905-1902 [15] we sought to provide a more robust estimate of VNTR mutation rates for *B. pseudomallei* by adding another isolate to the PSPE dataset. The genome-sequenced Bp305 (GenBank: AAYX00000000; http://www.ncbi.nlm.nih.gov/nuccore/AAYX00000000) was selected for this purpose.

One whole colony of Bp305 T_0_ (timepoint zero) was resuspended in 1 mL trypticase soy broth (TSB; Becton Dickinson) and 100 separate loopfuls were used for subculture onto 100 new trypticase soy agar (TSA) plates (T_1_) to establish 100 independent lineages. All Bp305 lineages were incubated at 37°C for 48 hours; in contrast, the PSPE lineages from Bp9905-1902 were subcultured at 24 h intervals. The difference in culture time between the two isolates was taken into account in all mutation rate estimates. A single, randomly chosen half-colony from each Bp305 lineage was subcultured on TSA to create T_2_ and incubated as described above. This process was iterated eight additional times until T_10_ was reached. The half-colonies not used for subculture were suspended in 1X TSB with 20% sterile glycerol and stored at −80°C to facilitate backtracking of mutation occurrence. DNA was extracted (as described in ‘DNA isolation’ above) from the original T_0_ suspension and all one hundred T_10_ isolates, and genotyped with MLVA. In instances where VNTR mutants were identified at T_10_ (i.e. VNTR sizes from T_10_ did not match data from T_0_), isolates from earlier timepoints of the corresponding lineage were subcultured and characterized by MLVA to ascertain whether the mutation was the result of a single- or multi-step mutation event (Table S1).

To calculate the number of generations in a 48 h colony, a randomly chosen T_0_ colony was subjected to a tenfold serial dilution, ranging from 10^−1^ to 10^−8^. One hundred microliters of each dilution was plated onto TSA and incubated for 48 h prior to colony counting. Based on these counts, approximately 1.69×10^8^ cells were present in one 48 h Bp305 colony, corresponding to 27.3 generations per 48 h colony, assuming each colony arose from a single cell. To determine per-locus mutation rates, the observed mutations at each locus for both Bp305 (this study) and Bp9905-1902 [15] were divided by the total number of generations for each isolate.

### Construction of phylogenies

For our study, a maximum parsimony method was chosen over other phylogenetic modeling methods (distance, maximum likelihood and Bayesian analysis) as maximum parsimony assumes a minimum number of mutational steps in estimating the evolutionary history, making it appropriate for modeling simple datasets such as clonally related *in vivo* populations [37]. The program eBURST v3 (http://eburst.mlst.net/) is a parsimony-based method that is widely used to determine the genetic relatedness of bacterial populations that have diverged over short evolutionary time spans based upon MLST data [25]. However, eBURST analysis is not limited to MLST and can be used with other datasets, such as MLVA data, when examining closely related isolates. Therefore, we used the ‘population snapshot’ feature of eBURST to identify founder genotypes and to define the population structure in the four *in vivo* within-host populations. eBURST predicts the founder genotype as the genotype with the greatest number of single-locus variants (and, where necessary, double-locus variants); often, the founder genotype is also numerically dominant [25].

Once the founder genotypes and population structure for the patients were determined using eBURST, we applied the general VNTR mutation model proposed by Vogler and co-workers [20],[22] to quantify the probability of mutations observed within both eBURST-derived and alternative maximally parsimonious phylogenies. The two *B. pseudomallei* PSPEs were used to determine mutation rates at loci mutated *in vivo*. In instances of multi-repeat mutations, the probabilities of single versus step-wise mutation events were compared using respective *in vitro* mutation rates. The products of the probabilities of each mutational event for all parsimonious phylogenies were determined, and the likelihood of each phylogenetic scenario was compared with an odds ratio, allowing the most likely phylogeny to be compared to eachalternative phylogeny [22],[38]. For each patient, the most likely phylogeny is presented; refer to Figures S1 and S2 for alternative phylogenies for P19 and P45, respectively.

## Supporting Information

Figure S1 Comparison of alternative *in vivo Burkholderia pseudomallei* population phylogenies for P19. Using *in vitro* variable-number tandem repeat (VNTR) mutation rates (Figure 2 and Table S2) to model the probability of *in vivo* mutations, phylogeny A was found to be more likely than alternative phylogenies B, C and D. However, the odds ratios for the alternative phylogenies were small, between 3.87 and 15, so we considered these four phylogenies to be approximately equally parsimonious. Although it has the highest odds ratio, phylogeny D best reflects patterns of *in vivo* evolution in this patient according to mutational backtracking. In addition, phylogeny D does not relate the 1764k-1 and 1764k-2 or 20k-1 and 20k-2 genotypes, consistent with these genotypes occurring independently at different tissue sites. Asterisks indicate the founder genotype; colors and circle sizes are described in Figure 1.
(0.41 MB DOC)

Figure S2 Comparison of alternative *in vivo Burkholderia pseudomallei* population phylogenies for P45. Unlike in P19 (Figure S1), alternative phylogenies for P45 contain differences in the number of character-state changes and thus were constructed without tree length restriction. In panels B and C, 2050k-3 was modeled as multi-step mutations, allowing a greater number of permissible character state changes compared with phylogeny A. Using *in vitro* variable-number tandem repeat (VNTR) mutation rates (Figure 2 and Table S2) to model the probability of all *in vivo* mutations, phylogeny A was found to be more likely than the alternative phylogenies (panels B and C), with odds ratios for the alternative phylogenies of 176 and 3,110,404, respectively. Consistent with this ranking, phylogeny A most accurately reflects the *in vivo* evolution as theoretical, intermediate genotypes (represented by a solid black square) from phylogenies B and C were not observed in our isolate set. Multi-step mutants of 3152k-8 were not modeled in P45 due to the highly unlikely possibility of multi-step mutations contributing to this mutation state (see Table 1 and Figure 2 for further explanation), which was in concordance with mutational backtracking that showed this mutation most probably occurred in a single step. Asterisks indicate the founder genotype; colors and circle sizes are described in Figure 1.
(0.32 MB DOC)

Table S1 Mutational backtracking of *Burkholderia pseudomallei* strain 305 (Bp305) lineages from a parallel serial passage experiment. One hundred Bp305 lineages were examined at timepoint 10 (T10) using multilocus variable-number tandem repeat analysis (MLVA). Thirty lineages possessed MLVA mutants at T10. In these lineages, Bp305 from previous timepoints were characterized by MLVA to identify the timepoint and mechanism of mutation (i.e. single-step or multi-step). Fields marked with ‘---’ represent data points that were not analyzed by MLVA. *DNA extractions and MLVA typing for timepoints T0 and T10 were conducted at the time of passage. For timepoints T1-T9, an additional subculture was carried out prior to MLVA genotyping. These genotypes could represent mutations that occurred following this additional passage step.
(0.55 MB DOC)

Table S2 *In vivo* variable-number tandem repeat (VNTR) mutations observed in the four melioidosis patients, their probabilities of occurrence and the theoretical number of generations for observing those mutations. ^a^Calculated using general VNTR mutation model describing the expected geometric distribution of VNTR mutation types: P(X = n) = p(1-p)n-1 [20] where p is the probability of a single-repeat mutation based upon observed data (74.3%), n is the number of repeats involved in the mutation, and P is the relative probability of a mutation involving n number of repeats. ^b^μ, observed probability of a given mutation at a specific locus. ^c^CI (confidence interval), calculated based upon Poisson distribution, describes the upper and lower generations required to observe a given mutation with 95% confidence [20],[22].
(0.04 MB DOC)
