# Supplementary material for: Within-Host Evolution of Burkholderia pseudomallei in Four Cases of Acute Melioidosis
Source: PLoS Pathog. 2010 Jan 15;6(1):e1000725. doi: 10.1371/journal.ppat.1000725 (PMC2799673; doi:10.1371/journal.ppat.1000725)
Supplement: Figure S1 — Comparison of alternative in vivo Burkholderia pseudomallei population phylogenies for P19. Using in vitro variable-number tandem repeat (VNTR) mutation rates (Figure 2 and Table S2) to model the probability of in vivo mutations, phylogeny A was found to be more likely than alternative phylogenies B, C and D. However, the odds ratios for the alternative phylogenies were small, between 3.87 and 15, so we considered these four phylogenies to be approximately equally parsimonious. Although it has the highest odds ratio, phylogeny D best reflects patterns of in vivo evolution in this patient according to mutational backtracking. In addition, phylogeny D does not relate the 1764k-1 and 1764k-2 or 20k-1 and 20k-2 genotypes, consistent with these genotypes occurring independently at different tissue sites. Asterisks indicate the founder genotype; colors and circle sizes are described in Figure 1. (0.41 MB DOC) [file ppat.1000725.s001.doc]

## 
