# Supplementary material for: Within-Host Evolution of Burkholderia pseudomallei in Four Cases of Acute Melioidosis
Source: PLoS Pathog. 2010 Jan 15;6(1):e1000725. doi: 10.1371/journal.ppat.1000725 (PMC2799673; doi:10.1371/journal.ppat.1000725)
Supplement: Table S1 — Mutational backtracking of Burkholderia pseudomallei strain 305 (Bp305) lineages from a parallel serial passage experiment. One hundred Bp305 lineages were examined at timepoint 10 (T10) using multilocus variable-number tandem repeat analysis (MLVA). Thirty lineages possessed MLVA mutants at T10. In these lineages, Bp305 from previous timepoints were characterized by MLVA to identify the timepoint and mechanism of mutation (i.e. single-step or multi-step). Fields marked with ‘---’ represent data points that were not analyzed by MLVA. *DNA extractions and MLVA typing for timepoints T0 and T10 were conducted at the time of passage. For timepoints T1-T9, an additional subculture was carried out prior to MLVA genotyping. These genotypes could represent mutations that occurred following this additional passage step. (0.55 MB DOC) [file ppat.1000725.s003.doc]

|  | VNTR locus | | | | | Comments | **Key** | |  |
| --- | --- | --- | --- | --- | --- | --- | --- | --- | --- |
|  | **2065k** | **2050k** | **3152k** | **2170k** | **1764k** |  | single repeat insertion |  |  |
| No. repeats in Bp305 T_0_ (original strain) | 11 | 18 | 17 | 25 | 19 |  | single repeat deletion |  |  |
|  |  |  |  |  |  |  | 2x repeat deletion |  |  |
| *Lineage 1* |  |  |  |  |  |  | 3x repeat deletion |  |  |
| Timepoint 1 (T_1_) | --- | --- | --- | --- | --- | **3152k:** mutated at T8 | 4x repeat deletion |  |  |
| T_2_ | --- | --- | --- | --- | --- |  | 5x repeat deletion |  |  |
| T_3_ | --- | --- | --- | --- | --- |  | 6x repeat deletion |  |  |
| T_4_ | --- | --- | --- | --- | --- |  | 7x repeat deletion |  |  |
| T_5_ | 11 | 18 | 17 | 25 | 19 |  | 8x repeat deletion |  |  |
| T_6_ | --- | --- | 17 | --- | --- |  | 9x repeat deletion |  |  |
| T_7_ | --- | --- | 17 | --- | --- |  |  |  |  |
| T_8_ | --- | --- | 18 | --- | --- |  |  |  |  |
| T_9_ | --- | --- | 18 | --- | --- |  |  |  |  |
| T_10_ | 11 | 18 | 18 | 25 | 19 |  |  |  |  |
| *Lineage 2* |  |  |  |  |  |  |  |  |  |
| T_1_ | --- | --- | --- | 25 | --- | **2170k:** mutated at T3 **2050k: s**ingle mutant at T5 caused by additional passage*. Two peaks at T8 caused by multiple genotypes in a single colony, a gene duplication event or 'stutter' artefact in PCR. Neither mutation was carried through to T10. |  |  |  |
| T_2_ | --- | --- | --- | 25 | --- |  |  |  |  |
| T_3_ | --- | --- | --- | 26 | --- |  |  |  |  |
| T_4_ | --- | --- | --- | 26 | --- |  |  |  |  |
| T_5_ | --- | 19 | 17 | 26 | 19 |  |  |  |  |
| T_6_ | --- | PCR-neg. | --- | --- | --- |  |  |  |  |
| T_7_ | --- | 18 | --- | --- | --- |  |  |  |  |
| T_8_ | --- | 17,18 | --- | --- | --- |  |  |  |  |
| T_9_ | --- | 18 | --- | --- | --- |  |  |  |  |
| T_10_ | 11 | 18 | 17 | 26 | 19 |  |  |  |  |
| *Lineage 3* |  |  |  |  |  |  |  |  |  |
| T_1_ | --- | --- | --- | --- | --- | **2050k:** mutated at T10 |  |  |  |
| T_2_ | --- | --- | --- | --- | --- |  |  |  |  |
| T_3_ | --- | --- | --- | --- | --- |  |  |  |  |
| T_4_ | --- | --- | --- | --- | --- |  |  |  |  |
| T_5_ | --- | 18 | 17 | 25 | 19 |  |  |  |  |
| T_6_ | --- | 18 | --- | --- | --- |  |  |  |  |
| T_7_ | --- | 18 | --- | --- | --- |  |  |  |  |
| T_8_ | --- | 18 | --- | --- | --- |  |  |  |  |
| T_9_ | --- | 18 | --- | --- | --- |  |  |  |  |
| T_10_ | 11 | 15 | 17 | 25 | 19 |  |  |  |  |
| *Lineage 4* |  |  |  |  |  |  |  |  |  |
| T_1_ | --- | 18 | --- | 25 | --- | **2050k:** mutated at T2 **2170k:** two peaks at T5 caused by multiple genotypes in a single colony, a gene duplication event or 'stutter' artefact in PCR. This mutation was not carried through to T10. |  |  |  |
| T_2_ | --- | 17 | --- | 25 | --- |  |  |  |  |
| T_3_ | --- | 17 | --- | 25 | --- |  |  |  |  |
| T_4_ | --- | 17 | --- | 25 | --- |  |  |  |  |
| T_5_ | --- | 17 | 17 | 23,25 | 19 |  |  |  |  |
| T_6_ | --- | --- | --- | --- | --- |  |  |  |  |
| T_7_ | --- | --- | --- | --- | --- |  |  |  |  |
| T_8_ | --- | --- | --- | --- | --- |  |  |  |  |
| T_9_ | --- | --- | --- | --- | --- |  |  |  |  |
| T_10_ | 11 | 17 | 17 | 25 | 19 |  |  |  |  |
| *Lineage 9* |  |  |  |  |  |  |  |  |  |
| T_1_ | --- | --- | --- | --- | --- | **2170k:** mutated at T9 |  |  |  |
| T_2_ | --- | --- | --- | --- | --- |  |  |  |  |
| T_3_ | --- | --- | --- | --- | --- |  |  |  |  |
| T_4_ | --- | --- | --- | --- | --- |  |  |  |  |
| T_5_ | --- | 18 | 17 | 25 | 19 |  |  |  |  |
| T_6_ | --- | --- | --- | --- | --- |  |  |  |  |
| T_7_ | --- | --- | --- | --- | --- |  |  |  |  |
| T_8_ | --- | --- | --- | --- | --- |  |  |  |  |
| T_9_ | --- | --- | --- | 24 | --- |  |  |  |  |
| T_10_ | 11 | 18 | 17 | 24 | 19 |  |  |  |  |
| *Lineage 16* |  |  |  |  |  |  |  |  |  |
| T_1_ | --- | --- | --- | --- | --- | **2170k:** mutated at T6, and again at T10 |  |  |  |
| T_2_ | --- | --- | --- | --- | --- |  |  |  |  |
| T_3_ | --- | --- | --- | --- | --- |  |  |  |  |
| T_4_ | --- | --- | --- | --- | --- |  |  |  |  |
| T_5_ | --- | 18 | --- | 25 | 19 |  |  |  |  |
| T_6_ | --- | --- | --- | 26 | --- |  |  |  |  |
| T_7_ | --- | --- | --- | 26 | --- |  |  |  |  |
| T_8_ | --- | --- | --- | 26 | --- |  |  |  |  |
| T_9_ | --- | --- | --- | 26 | --- |  |  |  |  |
| T_10_ | 11 | 18 | PCR neg. | 19 | 19 |  |  |  |  |
|  |  |  |  |  |  |  |  |  |  |
|  | VNTR locus | | | | | Comments | **Key** | |  |
|  | **2065k** | **2050k** | **3152k** | **2170k** | **1764k** |  | single repeat insertion |  |  |
| No. repeats in Bp305 T_0_ (original strain) | 11 | 18 | 17 | 25 | 19 |  | single repeat deletion |  |  |
|  |  |  |  |  |  |  | 2x repeat deletion |  |  |
| *Lineage 23* |  |  |  |  |  |  | 3x repeat deletion |  |  |
| T_1_ | --- | --- | --- | --- | --- | **2050k:** mutated at T7 | 4x repeat deletion |  |  |
| T_2_ | --- | --- | --- | --- | --- |  | 5x repeat deletion |  |  |
| T_3_ | --- | --- | --- | --- | --- |  | 6x repeat deletion |  |  |
| T_4_ | --- | --- | --- | --- | --- |  | 7x repeat deletion |  |  |
| T_5_ | --- | 18 | 17 | 25 | 19 |  | 8x repeat deletion |  |  |
| T_6_ | --- | 18 | --- | --- | --- |  | 9x repeat deletion |  |  |
| T_7_ | --- | 17 | --- | --- | --- |  |  |  |  |
| T_8_ | --- | 17 | --- | --- | --- |  |  |  |  |
| T_9_ | --- | 17 | --- | --- | --- |  |  |  |  |
| T_10_ | 11 | 17 | 17 | 25 | 19 |  |  |  |  |
| *Lineage 25* |  |  |  |  |  |  |  |  |  |
| T_1_ | --- | --- | --- | --- | --- | **2050k:** mutated at T8 |  |  |  |
| T_2_ | --- | --- | --- | --- | --- |  |  |  |  |
| T_3_ | --- | --- | --- | --- | --- |  |  |  |  |
| T_4_ | --- | --- | --- | --- | --- |  |  |  |  |
| T_5_ | --- | 18 | 17 | 25 | 19 |  |  |  |  |
| T_6_ | --- | 18 | --- | --- | --- |  |  |  |  |
| T_7_ | --- | 18 | --- | --- | --- |  |  |  |  |
| T_8_ | --- | 17 | --- | --- | --- |  |  |  |  |
| T_9_ | --- | 17 | --- | --- | --- |  |  |  |  |
| T_10_ | 11 | 17 | 17 | 25 | 19 |  |  |  |  |
| *Lineage 26* |  |  |  |  |  |  |  |  |  |
| T_1_ | --- | --- | 18 | --- | --- | **3152k:** mutated at T1 **2170k:** mutated at T6 |  |  |  |
| T_2_ | --- | --- | 18 | --- | --- |  |  |  |  |
| T_3_ | --- | --- | 18 | --- | --- |  |  |  |  |
| T_4_ | --- | --- | 18 | --- | --- |  |  |  |  |
| T_5_ | --- | 18 | 18 | 25 | 19 |  |  |  |  |
| T_6_ | --- | --- | --- | 24 | --- |  |  |  |  |
| T_7_ | --- | --- | --- | 24 | --- |  |  |  |  |
| T_8_ | --- | --- | --- | 24 | --- |  |  |  |  |
| T_9_ | --- | --- | --- | 24 | --- |  |  |  |  |
| T_10_ | 11 | 18 | 18 | 24 | 19 |  |  |  |  |
| *Lineage 27* |  |  |  |  |  |  |  |  |  |
| T_1_ | --- | --- | --- | --- | --- | **2170k:** mutated at T9 |  |  |  |
| T_2_ | --- | --- | --- | --- | --- |  |  |  |  |
| T_3_ | --- | --- | --- | --- | --- |  |  |  |  |
| T_4_ | --- | --- | --- | --- | --- |  |  |  |  |
| T_5_ | --- | 18 | 17 | 25 | 19 |  |  |  |  |
| T_6_ | --- | --- | --- | 25 | --- |  |  |  |  |
| T_7_ | --- | --- | --- | 25 | --- |  |  |  |  |
| T_8_ | --- | --- | --- | 25 | --- |  |  |  |  |
| T_9_ | --- | --- | --- | 26 | --- |  |  |  |  |
| T_10_ | 11 | 18 | 17 | 26 | 19 |  |  |  |  |
| *Lineage 36* |  |  |  |  |  |  |  |  |  |
| T_1_ | --- | --- | --- | --- | --- | **2170k:** mutated at T6 |  |  |  |
| T_2_ | --- | --- | --- | --- | --- |  |  |  |  |
| T_3_ | --- | --- | --- | --- | --- |  |  |  |  |
| T_4_ | --- | --- | --- | --- | --- |  |  |  |  |
| T_5_ | --- | 18 | 17 | 25 | 19 |  |  |  |  |
| T_6_ | --- | --- | --- | 24 | --- |  |  |  |  |
| T_7_ | --- | --- | --- | 24 | --- |  |  |  |  |
| T_8_ | --- | --- | --- | 24 | --- |  |  |  |  |
| T_9_ | --- | --- | --- | 24 | --- |  |  |  |  |
| T_10_ | 11 | 18 | 17 | 24 | 19 |  |  |  |  |
| *Lineage 38* |  |  |  |  |  |  |  |  |  |
| T_1_ | --- | --- | --- | 25 | --- | **2170k:** mutated at T5 |  |  |  |
| T_2_ | --- | --- | --- | 25 | --- |  |  |  |  |
| T_3_ | --- | --- | --- | 25 | --- |  |  |  |  |
| T_4_ | --- | --- | --- | 25 | --- |  |  |  |  |
| T_5_ | --- | 18 | 17 | 24 | 19 |  |  |  |  |
| T_6_ | --- | --- | --- | --- | --- |  |  |  |  |
| T_7_ | --- | --- | --- | --- | --- |  |  |  |  |
| T_8_ | --- | --- | --- | --- | --- |  |  |  |  |
| T_9_ | --- | --- | --- | --- | --- |  |  |  |  |
| T_10_ | 11 | 18 | 17 | 24 | 19 |  |  |  |  |
|  |  |  |  |  |  |  |  |  |  |
|  | VNTR locus | | | | | Comments | **Key** | |  |
|  | **2065k** | **2050k** | **3152k** | **2170k** | **1764k** |  | single repeat insertion |  |  |
| No. repeats in Bp305 T_0_ (original strain) | 11 | 18 | 17 | 25 | 19 |  | single repeat deletion |  |  |
|  |  |  |  |  |  |  | 2x repeat deletion |  |  |
| *Lineage 42* |  |  |  |  |  |  | 3x repeat deletion |  |  |
| T_1_ | --- | --- | --- | PCR neg. | --- | **2170k:** T1-T4 PCR negative, indicating possible insertion event. VNTR mutated at T5. Mutation at T7 due to additional passage*. | 4x repeat deletion |  |  |
| T_2_ | --- | --- | --- | PCR neg. | --- |  | 5x repeat deletion |  |  |
| T_3_ | --- | --- | --- | PCR neg. | --- |  | 6x repeat deletion |  |  |
| T_4_ | --- | --- | --- | PCR neg. | --- |  | 7x repeat deletion |  |  |
| T_5_ | --- | 18 | 17 | 17 | 19 |  | 8x repeat deletion |  |  |
| T_6_ | --- | --- | --- | 17 | --- |  | 9x repeat deletion |  |  |
| T_7_ | --- | --- | --- | 16 | --- |  |  |  |  |
| T_8_ | --- | --- | --- | 17 | --- |  |  |  |  |
| T_9_ | --- | --- | --- | 17 | --- |  |  |  |  |
| T_10_ | 11 | 18 | 17 | 17 | 19 |  |  |  |  |
| *Lineage 43* |  |  |  |  |  |  |  |  |  |
| T_1_ | 11 | --- | --- | --- | --- | **2065k:** mutated at T9 |  |  |  |
| T_2_ | --- | --- | --- | --- | --- |  |  |  |  |
| T_3_ | 11 | --- | --- | --- | --- |  |  |  |  |
| T_4_ | 11 | --- | --- | --- | --- |  |  |  |  |
| T_5_ | 11 | 18 | 17 | 25 | 19 |  |  |  |  |
| T_6_ | 11 | --- | --- | --- | --- |  |  |  |  |
| T_7_ | 11 | --- | --- | --- | --- |  |  |  |  |
| T_8_ | 11 | --- | --- | --- | --- |  |  |  |  |
| T_9_ | 12 | --- | --- | --- | --- |  |  |  |  |
| T_10_ | 12 | 18 | 17 | 25 | 19 |  |  |  |  |
| *Lineage 53* |  |  |  |  |  |  |  |  |  |
| T_1_ | --- | --- | --- | --- | --- | **2050k:** mutated at T8 |  |  |  |
| T_2_ | --- | --- | --- | --- | --- |  |  |  |  |
| T_3_ | --- | --- | --- | --- | --- |  |  |  |  |
| T_4_ | --- | --- | --- | --- | --- |  |  |  |  |
| T_5_ | --- | 18 | 17 | 25 | 19 |  |  |  |  |
| T_6_ | --- | 18 | --- | --- | --- |  |  |  |  |
| T_7_ | --- | 18 | --- | --- | --- |  |  |  |  |
| T_8_ | --- | 19 | --- | --- | --- |  |  |  |  |
| T_9_ | --- | 19 | --- | --- | --- |  |  |  |  |
| T_10_ | 11 | 19 | 17 | 25 | 19 |  |  |  |  |
| *Lineage 57* |  |  |  |  |  |  |  |  |  |
| T_1_ | --- | --- | --- | --- | --- | **2170k:** mutated at T10 |  |  |  |
| T_2_ | --- | --- | --- | --- | --- |  |  |  |  |
| T_3_ | --- | --- | --- | --- | --- |  |  |  |  |
| T_4_ | --- | --- | --- | --- | --- |  |  |  |  |
| T_5_ | --- | 18 | 17 | 25 | 19 |  |  |  |  |
| T_6_ | --- | --- | --- | 25 | --- |  |  |  |  |
| T_7_ | --- | --- | --- | 25 | --- |  |  |  |  |
| T_8_ | --- | --- | --- | 25 | --- |  |  |  |  |
| T_9_ | --- | --- | --- | 25 | --- |  |  |  |  |
| T_10_ | 11 | 18 | 17 | 26 | 19 |  |  |  |  |
| *Lineage 58* |  |  |  |  |  |  |  |  |  |
| T_1_ | --- | --- | --- | 18 | --- | **2170k:** mutated at T1 |  |  |  |
| T_2_ | --- | --- | --- | 18 | --- |  |  |  |  |
| T_3_ | --- | --- | --- | 18 | --- |  |  |  |  |
| T_4_ | --- | --- | --- | 18 | --- |  |  |  |  |
| T_5_ | --- | 18 | 17 | 18 | 19 |  |  |  |  |
| T_6_ | --- | --- | --- | --- | --- |  |  |  |  |
| T_7_ | --- | --- | --- | --- | --- |  |  |  |  |
| T_8_ | --- | --- | --- | --- | --- |  |  |  |  |
| T_9_ | --- | --- | --- | --- | --- |  |  |  |  |
| T_10_ | 11 | 18 | 17 | 18 | 19 |  |  |  |  |
| *Lineage 60* |  |  |  |  |  |  |  |  |  |
| T_1_ | --- | --- | --- | 22 | --- | **2170k:** mutated T1 |  |  |  |
| T_2_ | --- | --- | --- | 22 | --- |  |  |  |  |
| T_3_ | --- | --- | --- | 22 | --- |  |  |  |  |
| T_4_ | --- | --- | --- | 22 | --- |  |  |  |  |
| T_5_ | --- | 18 | 17 | 22 | 19 |  |  |  |  |
| T_6_ | --- | --- | --- | --- | --- |  |  |  |  |
| T_7_ | --- | --- | --- | --- | --- |  |  |  |  |
| T_8_ | --- | --- | --- | --- | --- |  |  |  |  |
| T_9_ | --- | --- | --- | --- | --- |  |  |  |  |
| T_10_ | 11 | 18 | 17 | 22 | 19 |  |  |  |  |
|  |  |  |  |  |  |  |  |  |  |
|  | VNTR locus | | | | | Comments | **Key** | |  |
|  | **2065k** | **2050k** | **3152k** | **2170k** | **1764k** |  | single repeat insertion |  |  |
| No. repeats in Bp305 T_0_ (original strain) | 11 | 18 | 17 | 25 | 19 |  | single repeat deletion |  |  |
|  |  |  |  |  |  |  | 2x repeat deletion |  |  |
| *Lineage 67* |  |  |  |  |  |  | 3x repeat deletion |  |  |
| T_1_ | --- | --- | --- | --- | --- | **3152k:** mutated at T7 | 4x repeat deletion |  |  |
| T_2_ | --- | --- | --- | --- | --- |  | 5x repeat deletion |  |  |
| T_3_ | --- | --- | --- | --- | --- |  | 6x repeat deletion |  |  |
| T_4_ | --- | --- | --- | --- | --- |  | 7x repeat deletion |  |  |
| T_5_ | --- | 18 | 17 | 25 | 19 |  | 8x repeat deletion |  |  |
| T_6_ | --- | --- | 17 | --- | --- |  | 9x repeat deletion |  |  |
| T_7_ | --- | --- | 14 | --- | --- |  |  |  |  |
| T_8_ | --- | --- | 14 | --- | --- |  |  |  |  |
| T_9_ | --- | --- | 14 | --- | --- |  |  |  |  |
| T_10_ | 11 | 18 | 14 | 25 | 19 |  |  |  |  |
| *Lineage 78* |  |  |  |  |  |  |  |  |  |
| T_1_ | --- | --- | --- | 25 | --- | **2170k:** mutated at T3 |  |  |  |
| T_2_ | --- | --- | --- | 25 | --- |  |  |  |  |
| T_3_ | --- | --- | --- | 16 | --- |  |  |  |  |
| T_4_ | --- | --- | --- | 16 | --- |  |  |  |  |
| T_5_ | --- | 18 | 17 | 16 | 19 |  |  |  |  |
| T_6_ | --- | --- | --- | --- | --- |  |  |  |  |
| T_7_ | --- | --- | --- | --- | --- |  |  |  |  |
| T_8_ | --- | --- | --- | --- | --- |  |  |  |  |
| T_9_ | --- | --- | --- | --- | --- |  |  |  |  |
| T_10_ | 11 | 18 | 17 | 16 | 19 |  |  |  |  |
| *Lineage 81* |  |  |  |  |  |  |  |  |  |
| T_1_ | --- | --- | --- | --- | --- | **2170k:** Two peaks at T8 and T10 caused by multiple genotypes in a single colony, a gene duplication event or 'stutter' artefact in PCR. The mutation at T10 was considered a deletion event in the current study. |  |  |  |
| T_2_ | --- | --- | --- | --- | --- |  |  |  |  |
| T_3_ | --- | --- | --- | --- | --- |  |  |  |  |
| T_4_ | --- | --- | --- | --- | --- |  |  |  |  |
| T_5_ | --- | 18 | 17 | 25 | 19 |  |  |  |  |
| T_6_ | --- | --- | --- | 25 | --- |  |  |  |  |
| T_7_ | --- | --- | --- | 25 | --- |  |  |  |  |
| T_8_ | --- | --- | --- | 25,26 | --- |  |  |  |  |
| T_9_ | --- | --- | --- | 25 | --- |  |  |  |  |
| T_10_ | 11 | 18 | 17 | 18,25 | 19 |  |  |  |  |
| *Lineage 83* |  |  |  |  |  |  |  |  |  |
| T_1_ | --- | 18 | --- | --- | --- | **2050k:** mutated at T2 |  |  |  |
| T_2_ | --- | 16 | --- | --- | --- |  |  |  |  |
| T_3_ | --- | 16 | --- | --- | --- |  |  |  |  |
| T_4_ | --- | 16 | --- | --- | --- |  |  |  |  |
| T_5_ | --- | 16 | 17 | 25 | 19 |  |  |  |  |
| T_6_ | --- | --- | --- | --- | --- |  |  |  |  |
| T_7_ | --- | --- | --- | --- | --- |  |  |  |  |
| T_8_ | --- | --- | --- | --- | --- |  |  |  |  |
| T_9_ | --- | --- | --- | --- | --- |  |  |  |  |
| T_10_ | 11 | 16 | 17 | 25 | 19 |  |  |  |  |
| *Lineage 84* |  |  |  |  |  |  |  |  |  |
| T_1_ | --- | --- | --- | 25 | --- | **2170k:** mutated at T5 |  |  |  |
| T_2_ | --- | --- | --- | 25 | --- |  |  |  |  |
| T_3_ | --- | --- | --- | 25 | --- |  |  |  |  |
| T_4_ | --- | --- | --- | 25 | --- |  |  |  |  |
| T_5_ | --- | 18 | 17 | 24 | 19 |  |  |  |  |
| T_6_ | --- | --- | --- | --- | --- |  |  |  |  |
| T_7_ | --- | --- | --- | --- | --- |  |  |  |  |
| T_8_ | --- | --- | --- | --- | --- |  |  |  |  |
| T_9_ | --- | --- | --- | --- | --- |  |  |  |  |
| T_10_ | 11 | 18 | 17 | 24 | 19 |  |  |  |  |
| *Lineage 87* |  |  |  |  |  |  |  |  |  |
| T_1_ | --- | --- | --- | --- | --- | **1764k:** mutated at T9 |  |  |  |
| T_2_ | --- | --- | --- | --- | --- |  |  |  |  |
| T_3_ | --- | --- | --- | --- | --- |  |  |  |  |
| T_4_ | --- | --- | --- | --- | --- |  |  |  |  |
| T_5_ | --- | 18 | 17 | 25 | 19 |  |  |  |  |
| T_6_ | --- | --- | --- | --- | 19 |  |  |  |  |
| T_7_ | --- | --- | --- | --- | 19 |  |  |  |  |
| T_8_ | --- | --- | --- | --- | 19 |  |  |  |  |
| T_9_ | --- | --- | --- | --- | 14 |  |  |  |  |
| T_10_ | 11 | 18 | 17 | 25 | 14 |  |  |  |  |
|  |  |  |  |  |  |  |  |  |  |
|  | VNTR locus | | | | | Comments | **Key** | |  |
|  | **2065k** | **2050k** | **3152k** | **2170k** | **1764k** |  | single repeat insertion |  |  |
| No. repeats in Bp305 T_0_ (original strain) | 11 | 18 | 17 | 25 | 19 |  | single repeat deletion |  |  |
|  |  |  |  |  |  |  | 2x repeat deletion |  |  |
| *Lineage 90* |  |  |  |  |  |  | 3x repeat deletion |  |  |
| T_1_ | --- | --- | --- | 25 | --- | **2170k:** mutated at T2 | 4x repeat deletion |  |  |
| T_2_ | --- | --- | --- | 19 | --- |  | 5x repeat deletion |  |  |
| T_3_ | --- | --- | --- | 19 | --- |  | 6x repeat deletion |  |  |
| T_4_ | --- | --- | --- | 19 | --- |  | 7x repeat deletion |  |  |
| T_5_ | --- | 18 | 17 | 19 | 19 |  | 8x repeat deletion |  |  |
| T_6_ | --- | --- | --- | --- | --- |  | 9x repeat deletion |  |  |
| T_7_ | --- | --- | --- | --- | --- |  |  |  |  |
| T_8_ | --- | --- | --- | --- | --- |  |  |  |  |
| T_9_ | --- | --- | --- | --- | --- |  |  |  |  |
| T_10_ | 11 | 18 | 17 | 19 | 19 |  |  |  |  |
| *Lineage 91* |  |  |  |  |  |  |  |  |  |
| T_1_ | --- | --- | 17 | --- | --- | **3152k:** mutated at T5 |  |  |  |
| T_2_ | --- | --- | 17 | --- | --- |  |  |  |  |
| T_3_ | --- | --- | 17 | --- | --- |  |  |  |  |
| T_4_ | --- | --- | 17 | --- | --- |  |  |  |  |
| T_5_ | --- | 18 | 18 | 25 | 19 |  |  |  |  |
| T_6_ | --- | --- | --- | --- | --- |  |  |  |  |
| T_7_ | --- | --- | --- | --- | --- |  |  |  |  |
| T_8_ | --- | --- | --- | --- | --- |  |  |  |  |
| T_9_ | --- | --- | --- | --- | --- |  |  |  |  |
| T_10_ | 11 | 18 | 18 | 25 | 19 |  |  |  |  |
| *Lineage 93* |  |  |  |  |  |  |  |  |  |
| T_1_ | --- | 18 | --- | --- | --- | **2050k:** mutated at T5 |  |  |  |
| T_2_ | --- | 18 | --- | --- | --- |  |  |  |  |
| T_3_ | --- | 18 | --- | --- | --- |  |  |  |  |
| T_4_ | --- | 18 | --- | --- | --- |  |  |  |  |
| T_5_ | --- | 19 | 17 | 25 | 19 |  |  |  |  |
| T_6_ | --- | --- | --- | --- | --- |  |  |  |  |
| T_7_ | --- | --- | --- | --- | --- |  |  |  |  |
| T_8_ | --- | --- | --- | --- | --- |  |  |  |  |
| T_9_ | --- | --- | --- | --- | --- |  |  |  |  |
| T_10_ | 11 | 19 | 17 | 25 | 19 |  |  |  |  |
| *Lineage 94* |  |  |  |  |  |  |  |  |  |
| T_1_ | --- | 18 | --- | --- | --- | **2050k:** mutated at T10 |  |  |  |
| T_2_ | --- | 18 | --- | --- | --- |  |  |  |  |
| T_3_ | --- | 18 | --- | --- | --- |  |  |  |  |
| T_4_ | --- | 18 | --- | --- | --- |  |  |  |  |
| T_5_ | --- | --- | 17 | 25 | 19 |  |  |  |  |
| T_6_ | --- | 18 | --- | --- | --- |  |  |  |  |
| T_7_ | --- | 18 | --- | --- | --- |  |  |  |  |
| T_8_ | --- | 18 | --- | --- | --- |  |  |  |  |
| T_9_ | --- | 18 | --- | --- | --- |  |  |  |  |
| T_10_ | 11 | 19 | 17 | 25 | 19 |  |  |  |  |
| *Lineage 95* |  |  |  |  |  |  |  |  |  |
| T_1_ | --- | --- | --- | 25 | --- | **2170k:** Two peaks at T2 and T4 caused by multiple genotypes in a single colony, a gene duplication event or 'stutter' artefact in PCR. Single deletion event at T5 was carried through to T10. |  |  |  |
| T_2_ | --- | --- | --- | 17,25 | --- |  |  |  |  |
| T_3_ | --- | --- | --- | 17 | --- |  |  |  |  |
| T_4_ | --- | --- | --- | 17,24 | --- |  |  |  |  |
| T_5_ | --- | 18 | 17 | 24 | 19 |  |  |  |  |
| T_6_ | --- | --- | --- | --- | --- |  |  |  |  |
| T_7_ | --- | --- | --- | --- | --- |  |  |  |  |
| T_8_ | --- | --- | --- | --- | --- |  |  |  |  |
| T_9_ | --- | --- | --- | --- | --- |  |  |  |  |
| T_10_ | 11 | 18 | 17 | 24 | 19 |  |  |  |  |
| *Lineage 100* |  |  |  |  |  |  |  |  |  |
| T_1_ | --- | --- | --- | 25 | --- | **2170k:** mutated at T5 |  |  |  |
| T_2_ | --- | --- | --- | 25 | --- |  |  |  |  |
| T_3_ | --- | --- | --- | 25 | --- |  |  |  |  |
| T_4_ | --- | --- | --- | 25 | --- |  |  |  |  |
| T_5_ | --- | 18 | 17 | 23 | 19 |  |  |  |  |
| T_6_ | --- | --- | --- | --- | --- |  |  |  |  |
| T_7_ | --- | --- | --- | --- | --- |  |  |  |  |
| T_8_ | --- | --- | --- | --- | --- |  |  |  |  |
| T_9_ | --- | --- | --- | --- | --- |  |  |  |  |
| T_10_ | 11 | 18 | 17 | 23 | 19 |  |  |  |  |
