# Supplementary material for: Within-Host Evolution of Burkholderia pseudomallei in Four Cases of Acute Melioidosis
Source: PLoS Pathog. 2010 Jan 15;6(1):e1000725. doi: 10.1371/journal.ppat.1000725 (PMC2799673; doi:10.1371/journal.ppat.1000725)
Supplement: Table S2 — In vivo variable-number tandem repeat (VNTR) mutations observed in the four melioidosis patients, their probabilities of occurrence and the theoretical number of generations for observing those mutations. aCalculated using general VNTR mutation model describing the expected geometric distribution of VNTR mutation types: P(X = n) = p(1-p)n-1 [20] where p is the probability of a single-repeat mutation based upon observed data (74.3%), n is the number of repeats involved in the mutation, and P is the relative probability of a mutation involving n number of repeats. bμ, observed probability of a given mutation at a specific locus. cCI (confidence interval), calculated based upon Poisson distribution, describes the upper and lower generations required to observe a given mutation with 95% confidence [20],[22]. (0.04 MB DOC) [file ppat.1000725.s004.doc]

| **VNTR locus** | **Locus mutation rate** | **Repeat difference** | **Mutation frequency^a^** | **μ (observed *in vitro* locus mutation rate × mutation frequency)^b^** | **Generations** | | |
| --- | --- | --- | --- | --- | --- | --- | --- |
|  |  |  |  |  | **1/μ** | **Lower 95% CI^c^** | **Upper 95% CI^c^** |
| 20k | 3.02 x 10^-5^ | 1 | 0.7428571 | 2.24 x 10^-5^ | 4.46 x 10^4^ | 1143 | 239348 |
| 20k |  | 2 | 0.1910204 | 5.77 x 10^-6^ | 1.73 x 10^5^ | 4446 | 930802 |
| 933k | 3.66 x 10^-5^ | 1 | 0.7428571 | 2.72 x 10^-5^ | 3.68 x 10^4^ | 943 | 197494 |
| 1764k | 2.20 x 10^-5^ | 1 | 0.7428571 | 1.63 x 10^-5^ | 6.12 x 10^4^ | 2118 | 443456 |
| 1764k |  | 2 | 0.1910204 | 4.20 x 10^-6^ | 2.38 x 10^5^ | 6103 | 1277739 |
| 1788k | 2.20 x 10^-5^ | 1 | 0.7428571 | 1.63 x 10^-5^ | 6.12 x 10^4^ | 2118 | 443456 |
| 1934k | 2.40 x 10^-6^ | 1 | 0.7428571 | 1.78 x 10^-6^ | 5.61 x 10^5^ | 14387 | 3011815 |
| 2050k | 1.97 x 10^-4^ | 1 | 0.7428571 | 1.46 x 10^-4^ | 6.83 x 10^3^ | 175 | 36690 |
| 2050k |  | 3 | 0.0491195 | 9.68 x 10^-6^ | 1.03 x 10^5^ | 2651 | 554911 |
| 2170k | 4.71 x 10^-4^ | 1 | 0.7428571 | 3.50 x 10^-4^ | 2.86 x 10^3^ | 73 | 15345 |
| 2445k | 2.40 x 10^-6^ | 1 | 0.7428571 | 1.78 x 10^-6^ | 5.61 x 10^5^ | 14387 | 3011815 |
| 3152k | 1.31 x 10^-4^ | 1 | 0.7428571 | 9.73 x 10^-5^ | 1.03 x 10^4^ | 264 | 55176 |
| 3152k |  | 8 | 5.52 x 10^-5^ | 7.23 x 10^-9^ | 1.38 x 10^8^ | 3545536 | 7.40 x 10^8^ |
| 3652k | 2.40 x 10^-6^ | 1 | 0.7428571 | 1.78 x 10^-6^ | 5.61 x 10^5^ | 14387 | 3011815 |
